# The shifting phenological landscape: Within‐ and between‐species variation in leaf emergence in a mixed‐deciduous woodland

**DOI:** 10.1002/ece3.2718

**Published:** 2017-01-24

**Authors:** Ella F. Cole, Ben C. Sheldon

**Affiliations:** ^1^ Edward Grey InstituteDepartment of ZoologyUniversity of OxfordOxfordUK

**Keywords:** budburst date, individual variation, phenology, small spatial scale, spring timing

## Abstract

Many organisms rely on synchronizing the timing of their life‐history events with those of other trophic levels—known as phenological matching—for survival or successful reproduction. In temperate deciduous forests, the extent of matching with the budburst date of key tree species is of particular relevance for many herbivorous insects and, in turn, insectivorous birds. In order to understand the ecological and evolutionary forces operating in these systems, we require knowledge of the factors influencing leaf emergence of tree communities. However, little is known about how phenology at the level of individual trees varies across landscapes, or how consistent this spatial variation is between different tree species. Here, we use field observations, collected over 2 years, to characterize within‐ and between‐species differences in spring phenology for 825 trees of six species (*Quercus robur*,* Fraxinus excelsior*,* Fagus sylvatica*,* Betula pendula*,* Corylus avellana*, and *Acer pseudoplatanus*) in a 385‐ha woodland. We explore environmental predictors of individual variation in budburst date and bud development rate and establish how these phenological traits vary over space. Trees of all species showed markedly consistent individual differences in their budburst timing. Bud development rate also varied considerably between individuals and was repeatable in oak, beech, and sycamore. We identified multiple predictors of budburst date including altitude, local temperature, and soil type, but none were universal across species. Furthermore, we found no evidence for interspecific covariance of phenology over space within the woodland. These analyses suggest that phenological landscapes are highly complex, varying over small spatial scales both within and between species. Such spatial variation in vegetation phenology is likely to influence patterns of selection on phenology within populations of consumers. Knowledge of the factors shaping the phenological environments experienced by animals is therefore likely to be key in understanding how these evolutionary processes operate.

## 1 Introduction

Over recent decades, climate change has caused dramatic shifts in the timing of life‐history events of many organisms (Durant et al., 2007; Keenan, 2015; Parmesan & Yohe, 2003; Root et al., 2003), bringing a renewed interest in the study of plant phenology (Wolkovich et al., 2014). In temperate regions, the timing of leaf emergence in spring affects a wide variety of ecosystem processes and ecological interactions (Panchen et al., 2014). In deciduous habitats, for example, the timing of leaf emergence determines the availability of food for many herbivorous insect species (Coyle et al., 2010; Feeny, 1970) and, in turn, insectivorous birds (Perrins, 1991; Van Noordwijk et al., 1995). Any disruption to such trophic synchrony, caused by changes in vegetation phenology, could have far‐reaching consequences for community structure and population dynamics (Post et al., 2009; Thackeray et al., 2010, 2016). Recent research has demonstrated that species and populations can differ considerably in their phenological responses to climate change (Roberts et al., 2015; Thackeray et al., 2010), and this can lead to ecological mismatch between trophic levels (Both et al., 2009; Sagarin et al., 1999). Concerns about these disruptions to ecological interactions have increased the relevance of understanding the factors influencing spring leaf emergence in different plant species. Such knowledge is essential to understand how landscapes as a whole are responding to changing climates, which will, in turn, facilitate our understanding of how organisms inhabiting these dynamic environments cope with environmental change.

Timing of leaf emergence reflects a balance between the benefits of maximizing the growing season, and hence photosynthetic carbon uptake, and the costs of frost damage caused by leafing too early in the year (Bennie et al., 2010; Kramer et al., 2010). While the precise physiological mechanisms that control bud development are not well understood, for most temperate species, air temperature is the main driver triggering release of bud dormancy. Temperature cues include both increasing spring temperature (“forcing” cues) and chilling requirements, where trees must be exposed to sufficient cold temperatures to be released from dormancy (Linkosalo et al., 2006). The latter requirement is thought to act as a safeguard to prevent trees from budding prematurely in response to a warm period during winter (Perry, 1971). Once chilling requirements are met, trees require a certain amount of time above a critical temperature to stimulate bud development (Cannell & Smith, 1986). Hence in warm springs, trees usually come into bud earlier; in northern Europe, for example, an increase of 1°C in spring temperature can advance spring budburst by between 3 and 8 days (Karlsen et al., 2007).

While much work has focused on describing large‐scale temperature effects on phenology, research has been comparatively slow to explore the diversity of phenological responses observed across different species and environments (Panchen et al., 2014; Wolkovich et al., 2014). Considerable differences in spring phenology exist between species, even when individuals are exposed to the same conditions (Lechowicz, 1984). For example, sessile oak (*Quercus petraea*) and European beech (*Fagus sylvatica*) show marked differences in their sensitivity to spring temperature, advancing their budburst date by 7.26 and 2.03 days per degree Celsius increase, respectively (Vitasse et al., 2009). Interspecific differences in spring phenology are thought to be largely a result of variation in the warming and chilling requirements between species (Marchin et al., 2015), as well as variation in sensitivity to nontemperature cues such as photoperiod. Indeed in some species, budburst is primarily controlled by photoperiod, with sensitivity to temperature only developing once a critical day length has been reached time (Basler & Körner, 2012). Like chilling requirements, photoperiod sensitivity is thought to act as a safeguard against premature budding during periods of mild winter temperatures (Marchin et al., 2015). Differences in cue sensitivity between species can often be explained by differences in physiology or ecology. For example, ring‐porous species tend to leaf out later than diffuse‐porous species, as their larger xylem vessels make them more vulnerable to frost damage (Marchin et al., 2015). It has also been suggested that more opportunistic pioneer species will adopt a more risky phenological strategies, relying solely on temperature cues, whereas late successional species will be more conservative, using the safeguards of large chilling requirement or high photoperiod sensitivity (Körner & Basler, 2010). In addition to ecological factors, evolutionary history has been shown to explain significant interspecific variation in phenological response (Panchen et al., 2014; Willis et al., 2008). For example, one recent large‐scale analysis of over 1,600 woody species revealed a strong phylogenetic signal for leaf out dates (Panchen et al., 2014).

Within temperate plant comminutes, a large proportion of variation in budburst timing can be explained by interspecific differences, but considerable variation remains within species (Crawley & Akhteruzzaman, 1988; Hinks et al., 2015; Lechowicz, 1984; Wesołowski & Rowiński, 2006). Budburst of individual pedunculate oak trees (*Quercus robur*) within a single population have been shown to vary by more than 3 weeks—a similar range to that observed in population means between years (Crawley & Akhteruzzaman, 1988). Studies monitoring interspecific variation in phenology over large spatial scales have shown strong influences of latitude, elevation, and air temperature (e.g., Kramer, 1995, Ducousso et al. 1996), as well as effects of soil composition (Wielgolaski, 2001), humidity (Wielgolaski, 2001), and tree age (Ununger et al., 1988). Furthermore, studies comparing the phenology of individuals in close proximity to one another, or in comparable environments, have observed pronounced and consistent individual differences in budburst timing, suggestive of considerable genetic or early environmental effects (Crawley & Akhteruzzaman, 1988; Hinks et al., 2015; Wesołowski & Rowiński, 2006). However, it is unknown how these multiple factors contribute to explaining phenological variation at small spatial scales, over tens or hundreds of meters. Nor is it known whether species within the same communities show similar spatial patterns of phenological variation to one another.

The ability to describe and predict the phenologically heterogeneous landscapes experienced by individual organisms is likely to prove a useful tool in ecological and evolutionary research. Indeed, where vegetation phenology varies at a scale relevant to the movements of individuals (e.g., dispersal or foraging ranges), such knowledge is essential to understand the selective forces operating in these systems. For example, several insect species with limited dispersal have been shown to become locally adapted to specific host trees due to strong selection to match egg hatching with leaf budburst (Komatsu & Akimoto, 1995; Van Dongen et al., 1997). In addition to driving genetic structure in populations, spatial variation in vegetation phenology can shape community structure and dynamics, influencing both the species composition and relative abundance of insects on different host trees (Crawley & Akhteruzzaman, 1988). Knowledge of phenological landscapes can also provide insight into how animals adjust their phenology to match their environment, and how selection acts on this plasticity. Breeding great tits (*Parus major*), which are limited in the distance that they can travel from dependent offspring, have been shown to time their egg laying relative to oak budburst, and therefore the timing of the peak in caterpillar abundance, within the immediate vicinity of their nest (Hinks et al., 2015). Such studies require a good understanding of how vegetation phenology varies over small spatial scales, but research in this area is lacking.

Here, we explore how spring phenology varies both within and between the dominant tree species in a mixed‐deciduous woodland. We use field observations collected over two spring seasons to quantify date of budburst and rate of bud development for 825 trees of six species (pedunculate oak (*Quercus robur*), common ash (*Fraxinus excelsior*), European beech (*Fagus sylvatica*), silver birch (*Betula pendula*), common hazel (*Corylus avellana*), and sycamore maple (*Acer pseudoplatanus*)) in a 385‐ha contiguous wood. We test a range of environmental predictors of individual variation in spring leaf budburst and development rate and explore how these phenological traits vary over space.

## 2 Methods

### 2.1 Study site

This study was carried out at Wytham Woods (51°46′N, 1°20′W, National Grid Reference SP4608), a 385‐ha mixed‐deciduous woodland in Oxfordshire, UK. The canopy composition of the Wytham is primarily pedunculate oak (*Quercus robur*), common ash (*Fraxinus excelsior*), sycamore maple (*Acer pseudoplatanu*, European beech (*Fagus sylvatica*), and silver birch (*Betula pendula*), with an understory of common hazel (*Corylus avellana*), hawthorn (*Crataegus momgyna*), elder (*Sambucus niger*), and field maple (*Acer campestre*) (Perrins, 1965). Wytham Woods consists of a mosaic of habitat types that can be broadly categorized as (1) ancient seminatural woodland, (2) secondary regeneration, (3) nineteenth‐century broadleaf plantation, and (4) twentieth‐century plantation and is located on a hill (altitude range 60–170 m above sea level). Soil type varies across the site, with thin soil over corallian limestone at higher altitudes, and the lower slopes consisting of deeper clay soil and sandy soil (Savill et al., 2010).

### 2.2 Tree selection

We selected 825 individual trees of our six focal species (*Quercus robur*,* Fraxinus excelsior*,* Fagus sylvatica*,* Betula pendula*,* Corylus avellana*, and *Acer pseudoplatanus*), distributed across the woodland, in order to explore small‐scale individual variation in spring leaf budburst. Grid posts have been placed throughout Wytham on a 100 × 100 m grid, corresponding to points on the Ordnance Survey National Grid. We selected 200 of the 484 Wytham grid posts as sampling locations (see Fig. S1 for focal grid post locations). Site selection was influenced by the fact that this work was done as part of a wider project concerning phenological matching between trees, invertebrates, and birds (*Paridae* spps.).

We aimed to select one tree of each of our six focal tree species in the 100 m^2^ surrounding each of our 200 sampling locations (where the sample location was in the center of a 100 m × 100 m grid square). However, not all species were present in all areas of the woodland; therefore, the number of trees sampled per location ranged between two and six. Of the 200 locations, oaks was present at 196, ash at 195, beech at 71, birch at 96, hazel at 156, and sycamore at 111. We selected the nearest mature tree (i.e., not a sapling) of each species to the grid post, excluding trees that were obviously unhealthy (trees where more than half of the crown was dead). To avoid any phenological bias in sampled trees, we selected trees prior to leaf development (during March 2013). The diameter at breast height (dbh) of each tree was measured and the location determined using a handheld GPS (Garmin GPSMAP 62). All trees were individually marked with aluminum tree tags (25 mm timber tags, Stanton Hope Ltd) so that they could be easily relocated. Between the 2013 and 2014 spring seasons, 10 of the focal trees fell (due to particularly strong winds in winter 2013/4) or lost their tags (two beech, three birch, three hazel, and two sycamore).

### 2.3 Scoring of tree phenology

**Figure 1:**
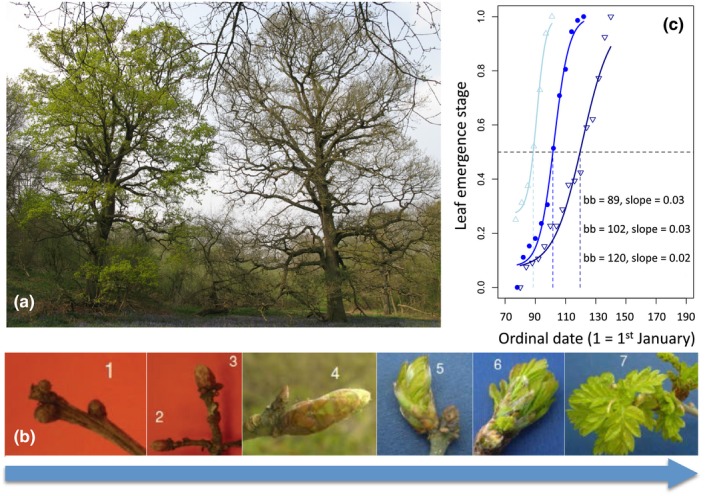
Scoring tree phenology: (a) two neighboring oak trees with contrasting leaf budburst timing, (b) seven‐stage phenological key used to score oak bud development, and (c) leaf emergence trajectory for three oak trees in 2014 (symbols show field observations of bud stage, the dashed line indicates the point of budburst of each tree). Leaf emergence stage, ranging from 0 (dormant buds) to 1 (fully emerged leaves, was measured via visual inspection of the buds throughout early spring (see Methods section). Budburst date (bb) and development rate (slope of curve at budburst) for the three example trajectories are shown in the figure

Observations of leaf development for the focal trees were conducted in 2013 and 2014 (March–May). Observations began when leaf buds started to swell and continued at 3‐day intervals until all shoots on the tree had developed small, unfolded leaves (see Hinks et al., 2015; Wesołowski & Rowiński, 2006). Developing buds were scored using a key of phenological stages ranging from “dormant buds” to “visible unfurled leaves.” We used a seven‐stage key for oaks developed by Hinks et al. (2015), where 1 = small dormant buds, 2 = larger, slightly elongated buds, 3 = larger, loosened greenish brown buds, 4 = further elongated buds with leaves starting to erupt (i.e., budburst), 5 = leaves emerging but still tight, 6 = leaves loosening and extending outwards, and 7 = leaves fully emerged and unfurled (see Figure 1b). We created additional five‐stage keys for each of the other species, where one corresponded to stage 1 on the oak scale (i.e., dormant bud), three corresponded to stage 4 on the oak scale (i.e., budburst), and five corresponded to stage 7 (i.e., leaves fully out). Stage 2 described elongated/swollen buds, and stage 4 described leaves emerging and extending outwards (see keys in supplementary material). These five‐stage keys were used because the five distinct stages could be easily observed for all six species. All scores were then normalized to have a range 0–1 and therefore were comparable across species. For each visit to a tree, a leaf development score was calculated by averaging visual scores for 12 sections of the canopy (three equal‐sized vertical sections, each split into four quarters). Any dead parts of the canopy were omitted from this calculation. All observations over the 2 years were carried out by five observers. The 825 trees were divided into nine “rounds,” which were split between three observers each year. Rounds were allocated so that each observer covered a similar range of habitats and altitudes. Before starting data collection, observers scored multiple trees together to minimize observer differences. They then met once a week throughout the scoring period to score a sample of trees together and compare measures to prevent divergence of scoring techniques. “Observer” was also controlled for in analyses (see below).

### 2.4 Predictors of individual variation in spring phenology

We tested a range of environmental predictors that have previously been linked to budburst timing: altitude and spring temperature (higher temperatures and lower elevations have been linked to early budburst, Wielgolaski, 2001), soil type (trees rooted in high moisture soil have been found to bud earlier, Wielgolaski, 2001), and habitat type, which is likely to influence microhabitat factors such as humidity levels (Wielgolaski, 2001) and also early environmental factors. We also test whether tree size, as a proxy for tree age, relates to budburst timing, as younger trees have been shown to bud earlier in the season (Ununger et al., 1988).

### 2.5 Temperature data

We collected local temperature data (accurate to ±0.5°C) at each of our 200 sampling locations throughout spring 2013 and 2014. A digital temperature logger (DS1923‐F5, HomeChip Ltd) attached to a plastic fob and suspended inside a plastic cup was hung on the north side of each grid post. Metallic foil trays (23 × 23 × 5 centimeters) were attached to the posts to further shade the temperature loggers. All loggers were approximately 1 m from ground level. Temperature was logged automatically every 30 min throughout April. To compare spring temperatures of 2013 and 2014 in the context of long‐term trends, we obtained long‐term temperature records for England from the Met Office (http://hadobs.metoffice.com/hadcet/).

### 2.6 Statistical analysis

#### 2.6.1 Budburst date and bud development rate

Bud development trajectories were calculated for each tree in each of the 2 years by fitting three‐parameter sigmoid functions to the time series of bud observations using nonlinear least squares models in R, version 3.2.2 (see Figure 1c, e.g., curves). Budburst date is generally defined as the stage in leaf development when green leaves first become visible, and has been used as a measure of leaf development for a range of deciduous trees in numerous studies (Hinks et al., 2015; Hunter & Lechowicz, 1992; Van Dongen et al., 1997; Watt & McFarlane, 1991; Wesołowski & Rowiński, 2006). For our study, budburst corresponded to stage four (of seven) in oaks and stage three (of five) in the other species. The budburst date and bud development rate for a given tree were calculated to be the date that the sigmoid curve passed through this budburst stage, and the gradient of the curve at this point, respectively (see Figure 1c). The repeatability of annual budburst timing and bud development rate of individual trees was estimated using the intraclass correlation coefficient. The amount of variance in budburst date within versus between species was estimated using linear mixed models.

#### 2.6.2 Predictors of individual‐level budburst date and bud development rate

We used spatial linear mixed models to explore potential predictors of intraspecific individual variation in budburst date and bud development rate, controlling for effects of spatial autocorrelation in the data using the R package spaMM (Rousset & Ferdy, 2014). Only repeatable phenological traits were tested (i.e., species that had either budburst date or development rate repeatabilities that were significant at the *p* = .05 level), and each species was analyzed separately. All models included fixed effects: tree size (dbh, centimeters), soil types (sand, clay, corallian limestone), habitat type (ancient seminatural woodland, secondary regeneration, nineteenth‐century broadleaf plantation, and twentieth‐century plantation), observer, and year. Measures of altitude (m) and spring temperature (April mean daily temperature, °C) were highly correlated; we therefore ran all models twice, once including altitude and once including temperature (in addition to the variables stated above). For the majority of models, neither altitude nor temperature were significant predictors, and the model outputs for the two models were broadly comparable; consequently, we present only the models including altitude. When altitude or temperature was significant, we present both effects. All models also included tree ID as a random effect. Spring temperature for a given tree was determined by extracting a measure of local temperature for each tree from an inverse distance‐weighted (IDW) interpolation of temperature data taken from the 200 fixed‐location sampling sites (using ArcMap 10.1). All continuous predictors were *z*‐transformed so that their effect sizes could be easily compared. All models (run using spaMM) included spatial data (X and Y coordinates) for each tree to correct for spatial autocorrelation. Support for variables was assessed based on breadth of confidence around the effect sizes; we discuss effects as statistically significant if they are more than twice the standard error around the estimate (Nakagawa & Cuthill, 2007).

In order to assess the extent to which budburst date covaried between different tree species across space, we conducted pairwise correlation analysis between pairs of species (15 possible correlations between six species, see Supplementary Table 1), which compared budburst date for species at the same sampling location across the woodland. All analyses were carried out in R, version 3.2.2 (http://www.R-project.org).

**Table 1:** Means and standard deviations of budburst dates and bud development rates 2013 and 2014 and between‐year repeatability estimates

Species	*N*	Budburst date					Bud development rate				
		2013		2014			2013		2014		
		Mean	*SD*	Mean	*SD*	Repeatability	Mean	*SD*	Mean	*SD*	Repeatability
*Quercus robur*	196	122	2.3	102	5.1	0.76***	0.050	0.014	0.036	0.009	0.38***
*Fraxinus excelsior*	195	127	3.2	130	5.7	0.57***	0.060	0.025	0.071	0.040	0.03
*Fagus sylvatica*	69	119	2.8	111	5.5	0.40***	0.059	0.019	0.046	0.013	0.47***
*Betula pendula*	93	114	2.6	96	4.5	0.74***	0.055	0.023	0.074	0.042	0.20^.^
*Corylus avellana*	153	107	2.0	88	6.0	0.65***	0.045	0.018	0.026	0.016	0.11
*Acer pseudoplatanus*	109	116	3.7	103	7.7	0.76***	0.047	0.017	0.036	0.010	0.41***

Budburst dates are given in ordinal dates (1 = 1st January).

***Repeatabilities significant at the *p* < .001 level, ^.^denotes repeatabilities where *p* < .1.

## 3 Results

### 3.1 Spring temperature during study period (2013 and 2014)

**Figure 2:**
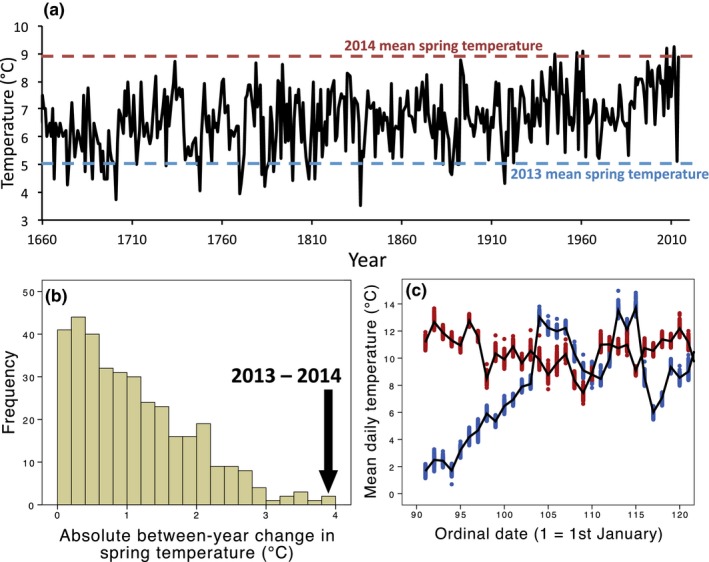
Climate data: (a) annual fluctuations in mean spring temperatures for England (1660–2014, °C), (b) absolute mean spring temperatures differences for consecutive pairs of years (1660–2014, °C, *N* = 355), (c) daily mean temperatures for Wytham Woods from 200 sampling locations (blue = 2013, red = 2014), black lines show woodland‐wide means for the 2 years. Long‐term temperature data shown in (a) and (b) is collected by the Hadley Centre Central Observatory

Average spring temperature (March–April) for England in 2013 was 5.1°C, making it the coldest spring for 91 years (see Figure 2a). In contrast, 2014 spring temperatures were above average (mean = 8.9°C), meaning that the absolute temperature difference between the two springs (3.8°C) was the second largest in the last 350 years, since records began (Figure 2b). In Wytham, this large between‐year temperature difference was present across the woodland until mid‐April (Figure 2c). Hence, the two years of this study contrasted markedly in terms of early spring temperature.

### 3.2 Interspecific variation in spring phenology

Spring bud development data for the 825 focal trees showed that five of the six tree species came into leaf earlier in 2014 than 2013 by between 1 and 3 weeks (difference in mean budburst date: oak (*N* = 196) = 20 days, hazel (*N* = 153) = 19 days, birch (*N* = 93) = 18 days, sycamore (*N* = 109) = 13 days, beech (*N* = 69) = 8 days, see Table 1). Ash alone showed little difference in mean budburst date between years, coming into leaf 3 days later in 2014 than 2013 (*N* = 195, Table 1). The order in which the six species came into leaf was significantly correlated between years (Pearson's *r* = .90, *p* = .016, *n* = 6). In 2013, hazel came into bud first, followed by birch, sycamore, beech, oak, and ash. In 2014, trees followed the same order with the exception of oak, which budburst just before sycamore (see Table 1). The relative speed of bud development was also significantly consistent between years for the six tree species (Pearson's correlation: *r* = .87, *p* = .023, *n* = 6), with ash, birch, and beech buds developing faster than oak, sycamore, and hazel buds.

### 3.3 Intraspecific variation in spring phenology

**Figure 3:**
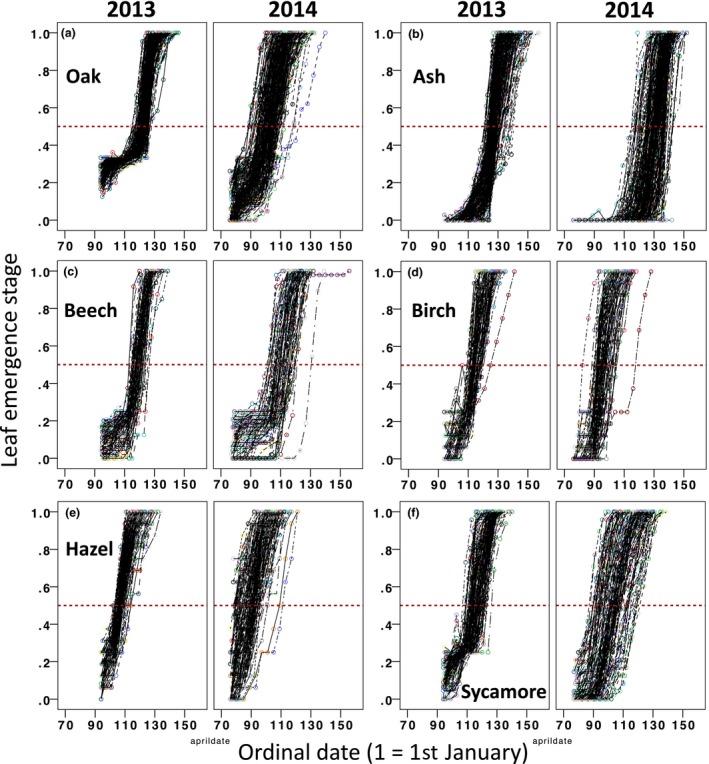
Raw budburst trajectories of individual trees in 2013 and 2014 for (a) oak (*N* = 196), (b) ash (*N* = 195), (c) beech (*N* = 71), (d) birch (*N* = 96), (e) hazel (*N* = 156), and (f) sycamore (*N* = 111). Red dashed line indicates budburst

**Figure 4:**
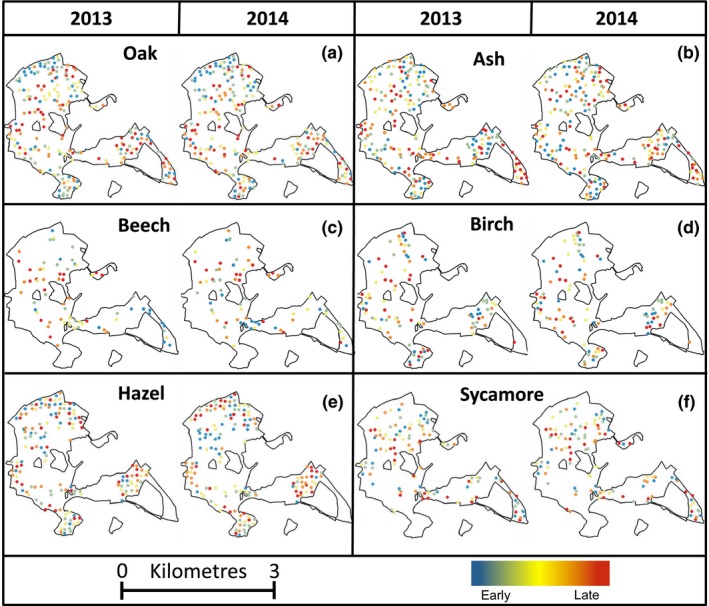
Maps of Wytham Woods showing the locations and budburst timing of all trees in 2013 and 2014 for (a) oak (*N* = 196), (b) ash (*N* = 195), (c) beech (*N* = 71), (d) birch (*N* = 96), (e) hazel (*N* = 156), (f) sycamore (*N* = 111). Marker color indicates ranked budburst date binned into five quartiles (blue–red denotes early–late)

All six species showed considerable individual variation in budburst date across the woodland (see Figures 3 and 4). In all species, individual trees were significantly repeatable in their relative budburst timing between years, with sycamore and oak showing the highest consistency and beech the lowest (*r* = .76, .76, .74, .65, .57, and .40 for sycamore, oak, birch, hazel, ash, and beech, respectively). Although individual trees were consistent in their timing relative to conspecifics within the woodland, intraspecific variability in budburst differed considerably between years, with all species showing greater spread in phenology in 2014 than 2013 (see Figure 3 and Table 1). Because interspecific variation in budburst was also greater in 2014 (see Table 1), the amount of variance within versus between species was similar across years (2013: between species = 86.1%, within species = 13.9%; 2014: between species = 86.0%, within species = 14.0%). Individual bud development rate was significantly repeatable in oak, beech, and sycamore, but not ash, birch, or hazel (see Table 1). Individual trees that came into bud later in the season had significantly faster bud development among ash, birch, and hazel (LMM: ash: *t* = 3.08 *p* = .002, *n* = 386; birch: *t* = 3.39 *p* = .001, *n* = 184; hazel: *t* = 10.55, *p* < .001, *n* = 281), but not among oak, beech, and sycamore (oak: *t* = 1.27 *p* = .21, *n* = 386; beech: *t* = 0.81 *p* = .424, *n* = 140; sycamore: *t* = 1.342, *p* = .182, *n* = 218).

### 3.4 Predictors of intraspecific variation in spring phenology

Among oak trees, individuals at higher altitudes had later budburst dates (see Table 2a); this effect was not solely driven by local temperature differences, as altitude was a stronger predictor of budburst date than local temperature (altitude: *t* = 3.17, β **±** *SE* = 1.48 ± 0.47; temperature: *t* = −2.14, β **±** *SE* = −3.82 ± 1.79). Local spring temperature was related to hazel budburst date, with trees in warmer areas of the woodland coming into leaf later than those in cooler areas (see Table 2e). Individual variation in budburst date in the remaining four species was unrelated to local temperature and altitude. Habitat type predicted timing among oaks, with trees in nineteenth‐century broadleaf plantation and secondary regenerated woodland coming into bud later than other habitat types (Table 2a). Tree size significantly predicted budburst variation in ash, beech, and hazel, with smaller ash and hazel trees coming into leaf sooner than large trees, and large beech trees coming into leaf sooner than smaller ones (Table 2). Finally, budburst date was related to soil type in sycamore, with trees coming into bud earliest in clay and sandy soil compared with corallian limestone (see Table 2f). These analyses did not identify any universal environmental predictors of small‐scale individual variation in budburst date across the six tree species (see Table 2). In agreement with this, we found no evidence that shared environment caused interspecific covariance in budburst across the woodland. The pairwise correlations comparing budburst date for species at the same sampling location across the woodland yielded no evidence for spatial covariance between species (see Table S1).

**Table 2:** Outputs from linear mixed models testing predictors of budburst date for individual trees

	Coefficient	SE	*t* value		Coefficient	*SE*	*t* value
(A) Oak				(B) Ash			
Intercept	**28.732**	**1.344**	**21.378**	Intercept	**37.411**	**1.414**	**26.461**
Altitude	**1.478**	**0.466**	**3.174**	Altitude	0.287	0.505	0.568
dbh	−0.193	0.240	−0.804	dbh	**0.779**	**0.266**	**2.923**
Soil: sand^d^	1.481	0.862	1.719	Soil: sand^d^	0.246	0.951	0.259
Soil clay^d^	1.669	1.135	1.471	Soil clay^d^	−0.762	1.236	−0.616
Habitat: 19th C plantation^a^	**2.854**	**1.021**	**2.795**	Habitat: 19th C plantation^a^	1.435	1.079	1.330
Habitat: regeneration^a^	**2.153**	**0.982**	**2.193**	Habitat: regeneration^a^	−0.721	1.022	−0.706
Habitat: ancient seminat^a^	0.812	0.860	0.943	Habitat: ancient seminat^a^	−1.060	0.877	−1.209
Observer BP^b^	**1.601**	**0.749**	**2.139**	Observer BP^b^	−0.490	0.851	−0.576
Observer SJC^b^	0.195	0.632	0.309	Observer SJC^b^	−0.045	0.813	−0.056
Observer SL^b^	**1.500**	**0.740**	**2.027**	Observer SL^b^	1.571	0.869	1.808
Observer ZD^b^	**2.373**	**0.871**	**2.725**	Observer ZD^b^	0.860	1.028	0.837
Year^c^	−**21.070**	**0.445**	−**47.357**	Year^c^	**2.732**	**0.577**	**4.737**
(C) Beech				(D) Birch			
Intercept	**30.596**	**1.576**	**19.412**	Intercept	**25.212**	**1.753**	**14.382**
Altitude	0.495	0.835	0.593	Altitude	0.395	0.615	0.643
dbh	−**1.424**	**0.472**	−**3.014**	dbh	−0.586	0.340	−1.725
Soil: sand^d^	0.103	1.171	0.088	Soil: sand^d^	−1.465	1.286	−1.140
Soil clay^d^	0.326	2.102	0.155	Soil clay^d^	−0.959	1.640	−0.585
Habitat: 19th C plantation^a^	−1.964	1.365	−1.438	Habitat: 19th C plantation^a^	−0.534	1.425	−0.375
Habitat: regeneration^a^	−0.927	1.640	−0.565	Habitat: regeneration^a^	0.874	1.259	0.695
Habitat: ancient seminat^a^	−1.731	1.233	−1.404	Habitat: ancient seminat^a^	0.478	0.915	0.523
Observer BP^b^	−2.880	1.624	−1.774	Observer BP^b^	−0.515	0.904	−0.570
Observer SJC^b^	−0.285	1.354	−0.211	Observer SJC^b^	−**2.514**	**0.782**	−**3.216**
Observer SL^b^	−0.631	1.421	−0.444	Observer SL^b^	0.329	0.976	0.337
Observer ZD^b^	−2.099	1.958	−1.072	Observer ZD^b^	−0.497	1.062	−0.468
Year^c^	−**7.659**	**1.068**	−**7.168**	Year^c^	−**17.626**	**0.553**	−**31.883**
(E) Hazel				(F) Sycamore			
Intercept	**21.253**	**3.151**	**6.745**	Intercept	**27.360**	**2.059**	**13.291**
Temperature	**5.423**	**2.619**	**2.071**	Altitude	−0.409	0.977	−0.418
dbh	**0.699**	**0.301**	**2.323**	dbh	−0.203	0.494	−0.412
Soil: sand^d^	0.596	0.997	0.598	Soil: sand^d^	−**4.155**	**1.696**	−**2.451**
Soil clay^d^	0.277	0.997	0.278	Soil clay^d^	−**5.239**	**2.419**	−**2.166**
Habitat: 19th C plantation^a^	−0.194	1.393	−0.139	Habitat: 19th C plantation^a^	0.363	1.701	0.213
Habitat: regeneration^a^	0.245	1.147	0.214	Habitat: regeneration^a^	1.083	1.815	0.597
Habitat: ancient seminat^a^	0.819	0.918	0.892	Habitat: ancient seminat^a^	−0.592	1.588	−0.373
Observer BP^b^	−0.466	0.973	−0.479	Observer BP^b^	0.808	1.881	0.430
Observer SJC^b^	−0.716	1.062	−0.674	Observer SJC^b^	−0.071	1.138	−0.062
Observer SL^b^	0.844	1.068	0.790	Observer SL^b^	2.834	1.644	1.724
Observer ZD^b^	0.266	1.237	0.215	Observer ZD^b^	0.429	2.040	0.210
Year^c^	−**29.391**	**5.222**	−**5.628**	Year^c^	−**12.739**	**0.788**	−**16.159**

All models contained the random effect “tree ID” and fixed effects: tree size (dbh), soil type (corallian limestone, sand, clay), habitat type (twentieth‐century plantation, nineteenth‐century broadleaf plantation, secondary regeneration, and ancient seminatural woodland), observer, year, and either altitude or ambient spring temperature (see Statistical Methods). All models correct for spatial autocorrelation by accounting for the spatial location of each tree (see Statistical Methods for further details).

a Twentieth‐century plantation set to zero.

b Observer AH set to zero.

c 2013 set to zero.

d Corallian limestone set to zero. Bold indicates the effects that are more than twice the standard error around the estimate.

Environmental predictors of bud development speed were only tested for oak, beech, and sycamore, as this phenological trait was not significantly repeatable for the remaining three species (see above). None of the environmental variables tested significantly predicted variation among oak and sycamore (Table 3a,c). In beech, budburst was significantly slower among large individuals and in areas of twentieth‐century plantation and ancient semi‐natural woodland (see Table 3b).

**Table 3:** Outputs from linear mixed models testing predictors of bud development rate for individual trees

	Coefficient	*SE*	*t* value
(A) Oak			
Intercept	**0.045**	**0.003**	**12.978**
Altitude	0.001	0.001	0.948
dbh	0.001	0.001	1.388
Soil: sand^d^	0.002	0.002	0.863
Soil clay^d^	0.005	0.003	1.606
Habitat: 19th C plantation^a^	0.002	0.003	0.610
Habitat: regeneration^a^	0.002	0.003	0.622
Habitat: ancient seminat^a^	0.004	0.002	1.611
Observer BP^b^	0.000	0.002	0.008
Observer SJC^b^	0.001	0.002	0.228
Observer SL^b^	**0.010**	**0.002**	**4.903**
Observer ZD^b^	0.004	0.003	1.315
Year^c^	**0.015**	**0.002**	**8.710**
(C) Beech			
Intercept	0.045	0.005	9.075
Altitude	0.003	0.003	1.013
dbh	−**0.006**	**0.002**	−**3.928**
Soil: sand^d^	−0.005	0.004	−1.386
Soil clay^d^	0.010	0.007	1.559
Habitat: 19th C plantation^a^	**0.011**	**0.004**	**2.412**
Habitat: regeneration^a^	**0.014**	**0.005**	**2.743**
Habitat: ancient seminat^a^	0.006	0.004	1.615
Observer BP^b^	0.006	0.005	1.233
Observer SJC^b^	**0.010**	**0.004**	**2.329**
Observer SL^b^	**0.009**	**0.004**	**2.036**
Observer ZD^b^	**0.023**	**0.006**	**3.643**
Year^c^	−**0.021**	**0.003**	−**6.018**
(E) Sycamore			
Intercept	**0.053**	**0.005**	**11.423**
Temperature	0.003	0.002	1.305
dbh	0.002	0.001	1.914
Soil: sand^d^	0.001	0.004	0.288
Soil clay^d^	0.007	0.005	1.349
Habitat: 19th C plantation^b^	0.004	0.004	1.113
Habitat: regeneration^a^	0.006	0.004	1.479
Habitat: ancient seminat^a^	0.000	0.004	0.025
Observer BP^a^	**0.011**	**0.004**	**2.554**
Observer SJC^b^	0.006	0.003	1.742
Observer SL^b^	**0.008**	**0.004**	**2.025**
Observer ZD^b^	0.001	0.005	0.246
Year^c^	**0.012**	**0.002**	**5.271**

All models contained the random effect “tree ID” and fixed effects: tree size (dbh), soil type (corallian limestone, sand, clay), habitat type (twentieth‐century plantation, nineteenth‐century broadleaf plantation, secondary regeneration, and ancient seminatural woodland), observer, year, and either altitude or ambient spring temperature (see Statistical Methods). All models correct for spatial autocorrelation by accounting for the spatial location of each tree (see Statistical Methods for further details).

a Twentieth‐century plantation set to zero.

b Observer AH set to zero.

c 2013 set to zero.

d Corallian limestone set to zero. Bold indicates the effects that are more than twice the standard error around the estimate.

## 4 Discussion

In recent years, there has been an increased effort to integrate perspectives across multiple disciplines in order to improve our ability to predict plant phenology across species, time, and space (reviewed in Wolkovich et al., 2014). Here, we aim to add to this effort by describing within‐ and between‐species phenological variation at a spatial scale largely neglected in existing literature. Using data collected over two consecutive—and meteorologically extreme—spring seasons, we explored environmental predictors of small‐scale individual variation in budburst date and bud development rate and establish how these phenological traits vary over space. We found that, within species, individual trees showed markedly consistent individual differences in their spring phenology across our 385‐ha study site. Environmental factors explained only a modest amount of this variation, and neither the predictors, nor the spatial patterns of variation, were consistent across species. Our findings suggest that, at the woodland level, phenological landscapes will be highly complex, varying over small spatial scales both within and between species.

The two spring seasons covered by our study were extremes in terms of spring temperature; in 2014, mean March–April temperature ranked the sixth warmest over the past 350 years for Central England, whereas 2013 saw the coldest March since 1917. In accordance with this dramatic difference in spring temperature, five of the six tree species came into leaf earlier in 2014 than 2013, but varied in their response, with oak showing the greatest plasticity with respect to temperature and beech the least (20 and 8 days of difference in budburst date between years, respectively). These results support findings from a study of phenological sensitivity to temperature along altitudinal gradients in seven deciduous tree species, which found oak to be the most sensitive and beech to be the least (Vitasse et al., 2009). Beech is known to be particularly sensitive to photoperiod and is thought to require long days before bud development can begin, even in particularly warm springs (Schaber & Badeck, 2003). It is becoming clear that tree species have different cue sensitivities and requirements and that this can cause the order and distribution of species budburst throughout spring to differ considerably between years (Roberts et al., 2015). Interestingly, we found that ash budburst date remained relatively constant in this pair of years, with the mean budburst occurring 3 days later in 2014 than 2013. This contrasts with studies demonstrating sensitivity to spring temperature in ash (Roberts et al., 2015; Vitasse et al., 2009) and therefore contradicts previous conclusions that sensitivity to global warming is stable for a given species (Vitasse et al., 2009).

Despite a growing literature on interspecific differences in spring timing, few studies have explored interspecific differences in within‐year variability in phenology. Recent work on a subarctic plant community found that early flowering species showed lower intraspecific variability in comparison with late flowering species, such that individual flowering times of early species were more closely tied to environmental predictors (Lessard‐Therrien et al., 2014). Here, we found that sycamore and ash consistently showed the most intraspecific variability in budburst date, and oak, beech, and birch consistently showed the least, but there was no link between budburst variance and budburst timing across species.

In agreement with previous work (Crawley & Akhteruzzaman, 1988; Hinks et al., 2015; Wesołowski & Rowiński, 2006), individual trees of all six species we monitored were repeatable in the relative order of their budburst between years. We also found that bud development rate was significantly repeatable in oak, beech, and sycamore, but not ash, birch, or hazel. Such marked individual differences among trees in close proximity (see Figures 1a and 4) suggest that genetic, early environmental, or developmental differences play a key role in determining spring phenology. Such differences appear to cause trees to interpret the same environmental cues in different ways to one another (Wesołowski & Rowiński, 2006). Spring phenology have been shown to be heritable in a range of species (Frewen et al., 2000; Scotti‐Saintagne et al., 2004), and common garden experiments suggest strong local adaptation (Chmura, 2006; Chmura & Rozkowski, 2002; Hannerz et al., 2003; Jensen & Hansen, 2008). Studies are beginning to identify candidate genes for spring bud development (Alberto et al., 2013; Derory et al., 2006; Morin et al., 2010; Scotti‐Saintagne et al., 2004; Ueno et al., 2013; Zohner & Renner, 2014); knowledge of genes that influence phenological traits will be essential for understanding the mechanisms underlying inherent individual differences in this trait, and the genetic structure of populations.

Despite individuals being consistent in their budburst timing relative to conspecifics within the woodland, we found that annual intraspecific variance in budburst differed considerably between years. All six species showed greater spread in budburst timing in 2014 than 2013 (standard deviations were between 1.7 and 3 times larger in 2014). This suggests that the extent to which intrinsic differences between individual trees influence spring phenology vary between years. While we lack the power to test drivers of this variation in the current study (having data for only two years), temperature would seem a likely candidate, given the marked annual difference observed in 2013 and 2014. To our knowledge, there has been little work exploring temporal or spatial variation in intraspecific budburst variance, despite its likely importance for higher trophic levels. High variability in vegetation phenology among neighboring trees is likely to increase the duration of the resource peak for organisms that feed on newly emerged leaves, and hence the food peak for consumers of these organisms. Increased variability in budburst date at small spatial scales therefore has the potential to relax selection on spring timing at higher trophic levels.

One of the aims of this study was to test whether observed variation in budburst within a single tree community could be explained by environment factors. The predictors we tested accounted for only a small amount of variation in budburst. We found little evidence for interspecific covariance in budburst across our study site. This suggests that there is no universal environmental predictor of individual variation in budburst date across species at this small spatial scale. Interestingly, despite the fact that temperature has often being found to be a strong predictor of large‐scale spatial variation in budburst timing (e.g., Chen et al., 2005; Kramer, 1995; Vitasse et al., 2009), this does not appear to be the case across the comparatively small spatial scales explored here. We found that temperature was a predictor of budburst date in hazel, with trees in colder areas, counter intuitively, coming into bud earlier than those in warmer areas. This perhaps suggests that trees in warmer areas may not receive sufficient chilling. Oak trees at higher altitudes came into bud later than those at lower altitudes, but this effect did not appear to be driven by local temperature differences, as temperature dropped out of the model at early stage. It should be noted that we used average temperature during April in this study; the window of temperature sensitivity triggering budburst in deciduous tree species is likely to start earlier in the year (e.g., window of thermal sensitivity in the UK for pedunculate oak, common ash, European beech, silver birch, and sycamore maple are estimated to be between the start of March and end of April, mid‐February to early May, the start of March and end of April, early March to mid‐April, and the start of February to early April, respectively, Tansey, 2016). Furthermore, winter chilling, which was not measured in present study, is known to be an important determinant of budburst in some species (Hadano et al., 2013; Richardson et al., 2006), although not in common ash, pedunculate oak, European beech, and sycamore maple (Tansey, 2016). Temperature data spanning winter and early spring are therefore needed to further explore how micro temperature differences may influence small‐scale patterns of budburst. We further note that the measures of budburst date and development rate used in this study were model‐derived estimates and therefore subject to a degree of uncertainty; this may therefore influence our ability to identify predictors of individual differences in phenology.

The findings from this study suggest that, when considering phenological variation over a small spatial scale, intraspecific differences caused by environment factors are largely overshadowed by inherent individual differences. This observation contrasts with data on variation over larger spatial scales, of tens or hundreds of kilometers, where strong environmental signals are usually present (Chen et al., 2005; Kramer, 1995; Vitasse et al., 2009). The question of how observed variation in life‐history traits, such as spring phenology, is dependent on the scale at which it is studied has long been a key issue in ecology (Levin, 1992). There is no single natural scale at which ecological phenomena should be studied, because organisms usually operate over a range of scales, across which there is often substantial environmental heterogeneity. The fact that there appears to be no obvious environmental proxies for budburst variation at the spatial scale we explored in this study presents a significant challenge for those aiming to describe and predict the phenology of tree communities. The ability to quantify these phenologically complex habitats is likely to be key in understanding how evolutionary processes operate on the animals inhabiting these environments. One promising approach for describing landscape‐level vegetation phenology is to use unmanned aerial vehicles (UAVs) to collect high‐resolution green‐up data. These devices are becoming increasingly affordable and have an extremely high spatial resolution (Chabot & Bird, 2013). As technology improves, these types of devices are likely to play an important role in the study of spatial ecology (Anderson & Gaston, 2013).

During recent years, the range of people interested in vegetation phenology has grown, as have the methods employed to study it (Polgar & Primack, 2011). Our perspective comes from an interest in the phenological landscape as it is experienced by higher trophic levels (e.g., Cole et al., 2015; Hinks et al., 2015). The analysis presented here demonstrates the complexity of phenological landscapes, when considered at a small spatial scale. At this resolution, spatial variation is driven largely by inherent individual differences rather than predictable environmental factors. More work is needed to understand how the relative importance of different drivers of phenological variation varies depending on the spatial scale being considered. The genetics of spring budburst timing is a rapidly developing field and likely to provide important insight into the mechanisms governing the distribution of different phenological phenotypes across space.

## Conflict of interest

None declared.
