# Supplementary material for: The shifting phenological landscape: Within‐ and between‐species variation in leaf emergence in a mixed‐deciduous woodland
Source: Ecol Evol. 2017 Jan 24;7(4):1135–47. doi: 10.1002/ece3.2718 (PMC5305997; doi:10.1002/ece3.2718)
Supplement: Supplementary file 1 [file ECE3-7-1135-s001.docx]

**Supplementary Material**

**Supplementary Figure 1.** Map of Wytham Woods showing the 200 sampling locations (black crosses) and 1020 long-term nestbox sites (grey circles).

**Supplementary Figure 2.** Five-stage phenological key used to score ash bud development, where (1) small dormant buds, (2) larger swollen buds, (3) loose buds, with leaves starting to erupt, (4) leaves obvious and extending outwards, and (5) leaves fully emerged. Images taken from Google images.


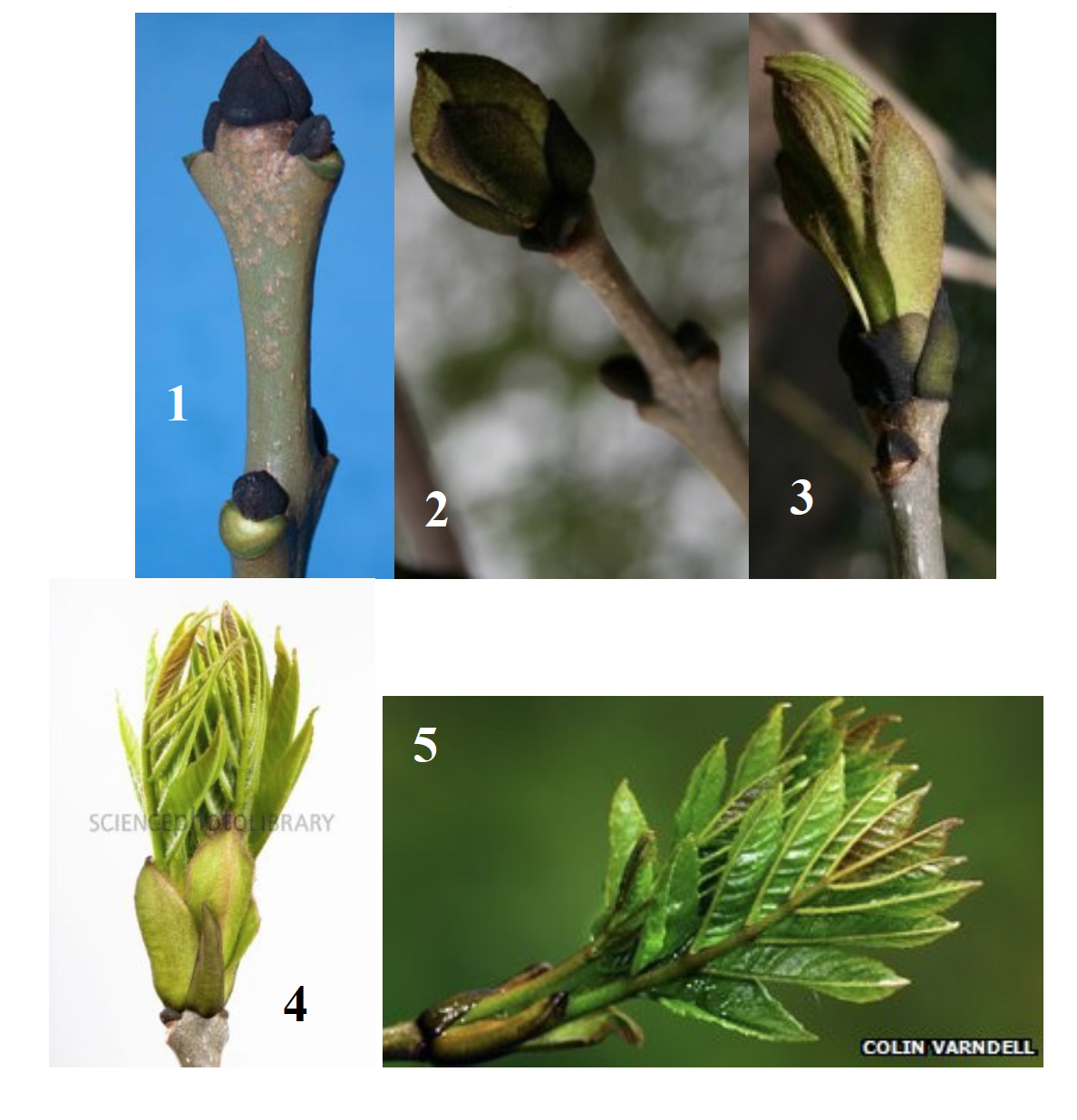


**Supplementary Figure 3.** Five-stage phenological key used to score beech bud development, where (1) small dormant buds, (2) larger swollen buds, (3) loose buds, with leaves starting to erupt, (4) leaves obvious and extending outwards, and (5) leaves fully emerged. Images taken from Google images.


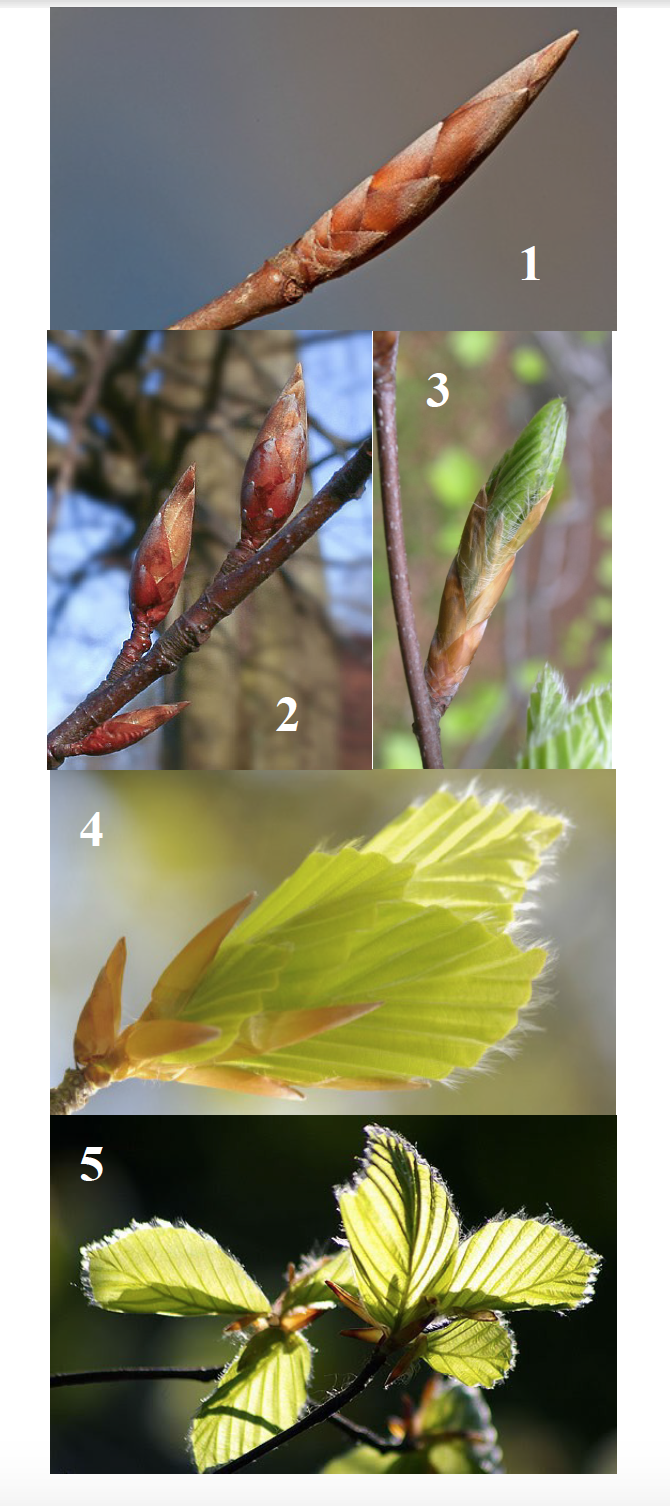


**Supplementary Figure 4.** Five-stage phenological key used to score birch bud development, where (1) small dormant buds, (2) larger swollen buds, (3) loose buds, with leaves starting to erupt, (4) leaves obvious and extending outwards, and (5) leaves fully emerged. Images taken from Google images.
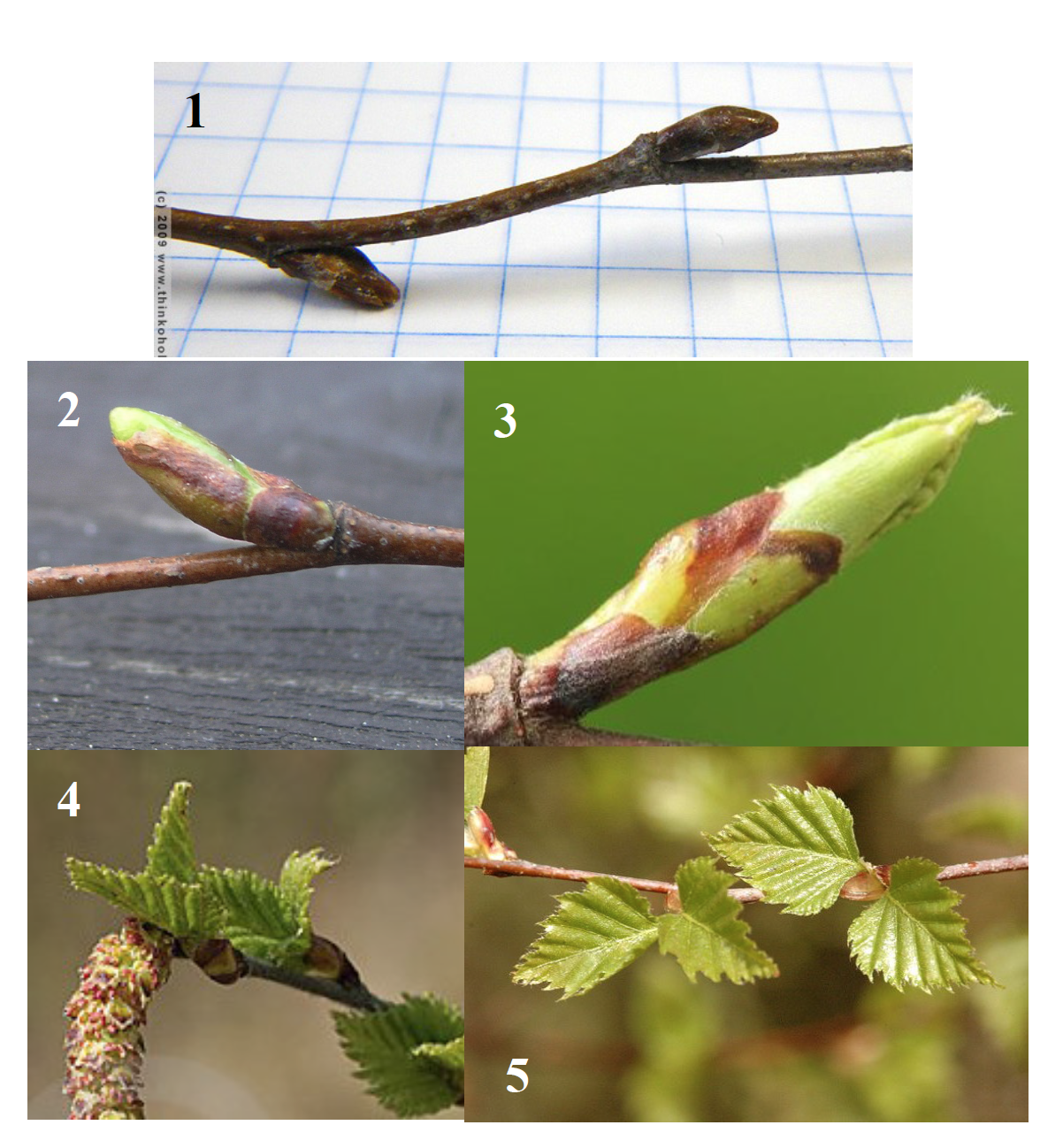


**Supplementary Figure 5.** Five-stage phenological key used to score hazel bud development, where (1) small dormant buds, (2) larger swollen buds, (3) loose buds, with leaves starting to erupt, (4) leaves obvious and extending outwards, and (5) leaves fully emerged. Images taken from Google images.


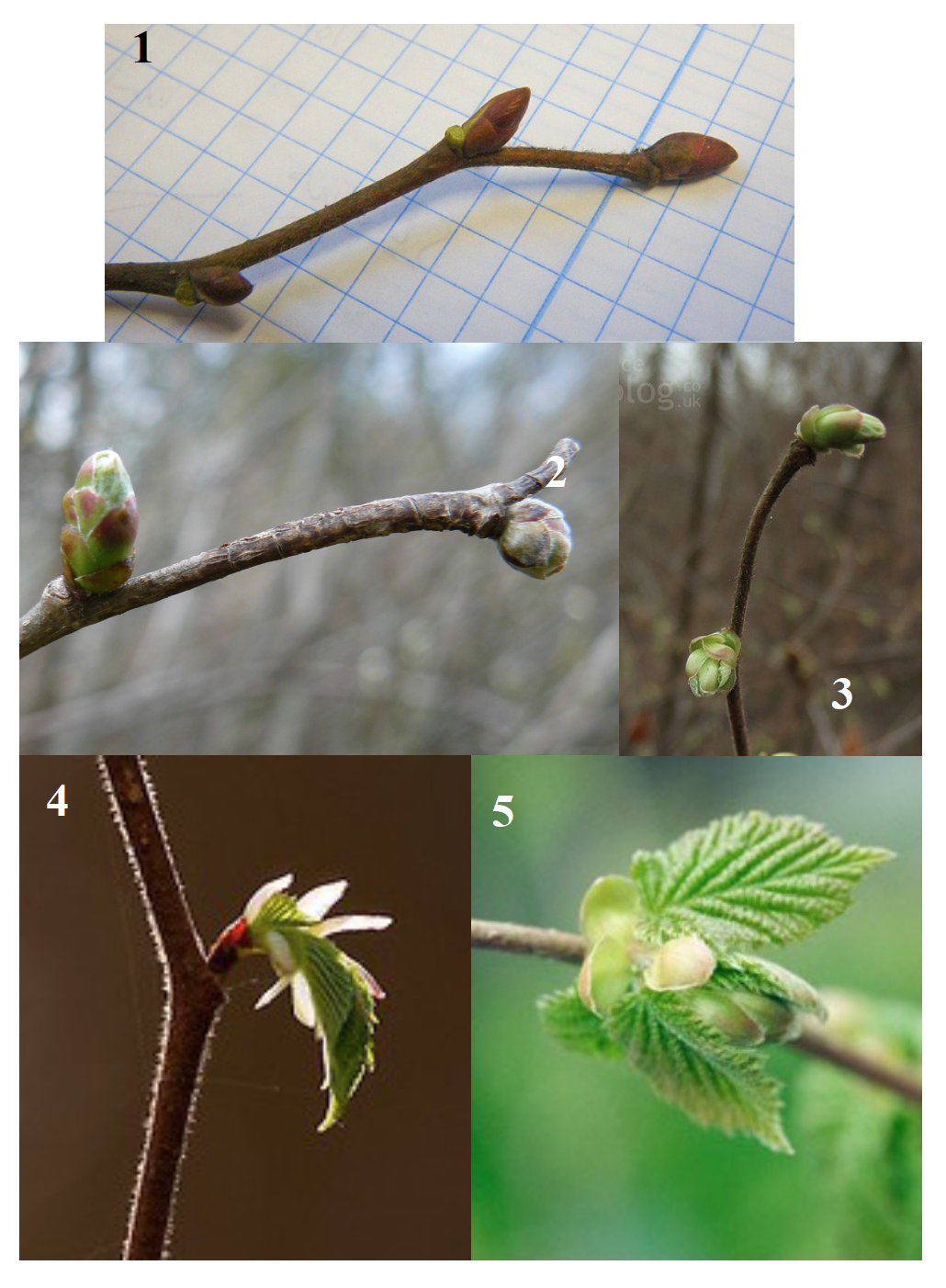


**Supplementary Figure 6.** Five-stage phenological key used to score sycamore development, where (1) small dormant buds, (2) larger swollen buds, (3) loose buds, with leaves starting to erupt, (4) leaves obvious and extending outwards, and (5) leaves fully emerged. Images taken from Google images.


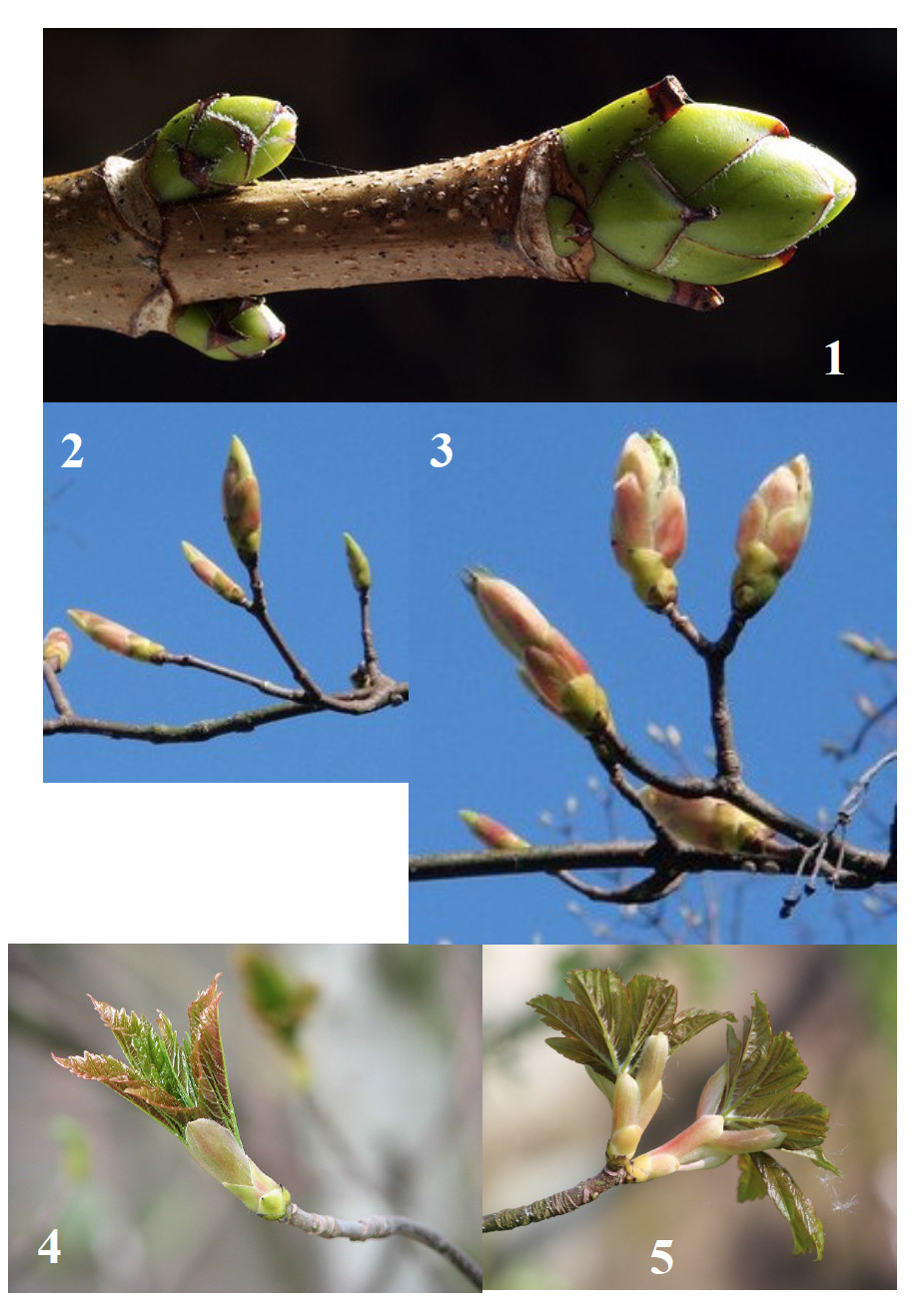


**Supplementary Table 1**. Matrices show correlation coefficients (r) between the budburst dates of the six different species across space and respective sample sizes (N = 200 locations).

| **(A) 2013** | *Quercus robur* | *Fraxinus excelsior* | *Fagus sylvatica* | *Betula pendula* | *Corylus avellana* | *Acer pseudoplatanus* |
| --- | --- | --- | --- | --- | --- | --- |
| *Quercus robur* |  | 0.14 | 0.01 | 0.14 | -0.02 | -0.01 |
| *Fraxinus excelsior* | *192* |  | -0.08 | 0.17 | 0.02 | 0.04 |
| *Fagus sylvatica* | *66* | *68* |  | 0.06 | 0.09 | -0.05 |
| *Betula pendula* | *91* | *92* | *29* |  | 0.06 | 0.12 |
| *Corylus avellana* | *151* | *149* | *38* | *82* |  | -0.18 |
| *Acer pseudoplatanus* | *106* | *104* | *54* | *44* | *72* |  |

| **(B) 2014** | *Quercus robur* | *Fraxinus excelsior* | *Fagus sylvatica* | *Betula pendula* | *Corylus avellana* | *Acer pseudoplatanus* |
| --- | --- | --- | --- | --- | --- | --- |
| *Quercus robur* |  | 0.05 | -0.12 | 0.18 | -0.02 | -0.09 |
| *Fraxinus excelsior* | *192* |  | -0.04 | 0.11 | -0.08 | 0.01 |
| *Fagus sylvatica* | *66* | *68* |  | 0.26 | -0.09 | 0.10 |
| *Betula pendula* | *91* | *92* | *29* |  | 0.03 | 0.14 |
| *Corylus avellana* | *151* | *149* | *38* | *82* |  | -0.16 |
| *Acer pseudoplatanus* | *106* | *104* | *54* | *44* | *72* |  |

Regression sample sizes are shown in the grey cells. None of the correlations were significant at the p = 0.05 level.
